# Prenatal Cannabis Use Disorder and Risk of Neurodevelopmental Disorders in Offspring: A Linked Data Cohort

**DOI:** 10.1192/j.eurpsy.2024.179

**Published:** 2024-08-27

**Authors:** A. W. Tadesse, B. A. Dachew, G. Ayano, K. Betts, R. Alati

**Affiliations:** ^1^School of Population Health, Curtin University, Perth, Australia; ^2^School of Population Health, Curtin University, Perth, Austria

## Abstract

**Introduction:**

Cannabis use has been increasing among women of reproductive age in the last few decades. In-utero cannabis exposure could be associated with an increased risk of neurodevelopmental disorders such as attention deficit hyperactivity disorder (ADHD), autism spectrum disorder (ASD), and intellectual disability (ID) during childhood and adolescence; however, existing evidence was generated based on maternal self-report of cannabis use in pregnancy. We conducted a large-scale with data linkage cohort study, in which both exposure and outcome of interests were confirmed using diagnostic tools, ICD-10-AM.

**Objectives:**

This study aimed to examine the association between prenatal cannabis use disorder (CUD) and neurodevelopmental disorders in offspring using a large-scale cohort study.

**Methods:**

We conducted an administrative health data-based cohort study of 222,569 mother-offspring pairs using linked data obtained from health registries in New South Wales (NSW), Australia. Data were drawn from the NSW Perinatal Data Collection (PDC), which included all live births in the Australian state of NSW between January 2003 and December 2005. These were linked with the NSW in-patient and ambulatory data collections for mothers and offspring. The prenatal cannabis use disorder (exposure) and neurodevelopmental disorders in offspring (outcomes of interest) were measured by using ICD-10-AM. Generalized linear regression with a binomial family model was used to explore the association. We also carried out a modification/interaction effect of low birth weight (LBW), smoking and premature births (PTB), which enhanced the methodological robustness of the study.

**Results:**

This study found that offspring from mothers with prenatal CUwD had a 98%, 94% and 46% increased risk of ADHD [aRR = 1.98: 95 % CI 1.36 – 2.88], ASD [aRR = 1.94: 95 % CI 1.34 – 2.82], and ID [aRR = 1.46: 95 % CI 1.01 – 2.63] compared to those non-exposed offspring, respectively. We observed a significant interaction effect between CUD during pregnancy and maternal smoking on the risk of childhood ADHD, ASD and ID [CUD*smoking: RR = 5.62: 95 % CI 3.77 – 8.39, RR = 2.72: 95 % CI 1.78 – 4.18, and RR = 2.84: 95 % CI 1.54 – 5.22, respectively]. Furthermore, we also found significant associations between PCUD and ADHD, ASD and ID when interacting with LBW, and PTB.

**Conclusions:**

Maternal prenatal CUD is associated with a higher risk of ADHD, ASD, and ID in offspring. The effect of maternal CUD on neurodevelopmental disorders was also found to be stronger when mothers also reported smoking during pregnancy, compared to the individual effects of cannabis use or smoking alone. The findings highlight the importance of implementing preventive strategies to reduce cannabis use in pregnancy.

**Disclosure of Interest:**

None Declared

